
JOURNAL INFORMATION
==============================
NLM Title Abbreviation: ScientificWorldJournal
Journal ID: ScientificWorldJournal
NLM Journal ID: 101131163
Journal ID: TSWJ

The Scientific World Journal

ISSN: 2356-6140
EISSN: 1537-744X
Publisher: Wiley

ARTICLE INFORMATION
==============================
PMCID: PMC5763768
PMID: 20526529
DOI: 10.1100/tsw.2010.93
Article ID: 603920
Article version: 1
Subjects: Clinical Image

Atrial Septal Aneurysm: A Major Diagnostic Clue for a Patent Foramen Ovale

Nia Amir M.
Gassanov Natig
Caglayan Evren
Er Fikret *
Department of Internal Medicine III, University of Cologne, Germany
*Fikret Er: fikret.er@uk-koeln.deAcademic Editor: Daniel Batlle

Publication date: 2010
Electronic publication date: 2010 Jun 1
Volume: 10
First page: 988
Last page: 989
Received 2010 Apr 11; Revised 2010 Apr 26; Accepted 2010 Apr 28
Copyright: Copyright © 2010 Amir M. Nia et al.
Copyright year: 2010
License: This is an open access article distributed under the Creative Commons Attribution License, which permits unrestricted use, distribution, and reproduction in any medium, provided the original work is properly cited.
License URL: https://creativecommons.org/licenses/by/3.0/

Keywords: patent foramen ovale, transthoracic echocardiography, transesophageal echocardiography, atrial septal aneurysm, migraine, interatrial septum

==============================
Clinical Image
*TheScientificWorldJOURNAL* (2010) 10, 988–989
ISSN 1537-744X; DOI 10.1100/tsw.2010.93

TheScientificWorldJOURNAL
www.thescientificworld.com

### Atrial Septal Aneurysm: A Major Diagnostic Clue for a Patent Foramen Ovale

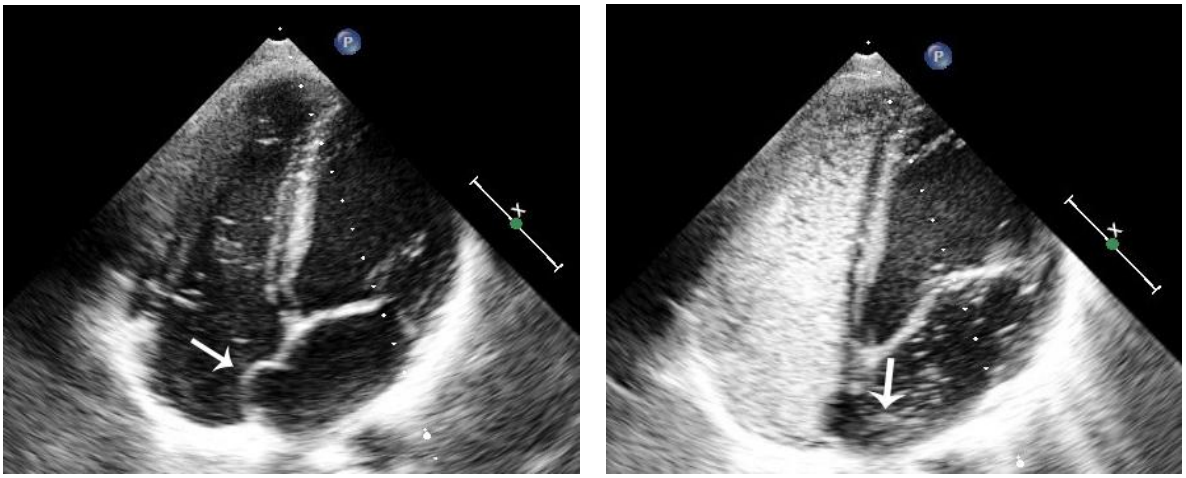

A B

**FIGURE 1.** (A) Transthoracic echocardiography imaging revealed a typical half-moon-shaped atrial septal aneurysm (arrow) in the apical four-chamber view. (B) Incompetence of the interatrial septum is seen during contrast bolus injection. Hereby transit of contrast-medium from right to left is clearly evident (arrow).

Amir M. Nia, Natig Gassanov, Evren Caglayan, and Fikret Er
*Department of Internal Medicine III, University of Cologne, Germany*

**E-mail**: Fikret.Er@uk-koeln.de

*Received April 11, 2010; Revised April 26, 2010; Accepted April 28, 2010; Published June 1, 2010*

**KEYWORDS**: patent foramen ovale, transthoracic echocardiography, transesophageal echocardiography, atrial septal aneurysm, migraine, interatrial septum

### CASE

Abnormalities of the atrial septum are relatively common and usually congenital in origin[1]. One prominent example is an atrial septal aneurysm (ASA), a saccular deformity of the interatrial septum. Most ASAs are associated with atrial septal defects (ASDs), usually exposed as a patent foramen ovale (PFO).

We describe a clinical case of a 23-year-old, healthy medical student who volunteered for a routine transthoracic echocardiography (TTE) as a part of the students' physical examination course. During the

*Corresponding author.
©2010 with author.
Published by TheScientificWorld; www.thescientificworld.com echocardiogram, an apical four-chamber view revealed an exaggerated mobility of the atrial septum, a classic sign of an ASA (Fig. 1; Panel A; Arrow).

The gold standard for diagnosing PFO is to perform a transesophageal echocardiography (TEE)[2]. In our case, given that the ASA was an incidental finding, the subject was asymptomatic, and the risks associated with performing a TEE, we used the TTE to prove interatrial septal permeability. Valsalva’s maneuver was performed to increase intrathoracic pressure, venous contrast medium was injected, and a clear passover of bubbles from the right to the left atrium was observed (Fig. 1; Panel B; Arrow). Thus, presence of a PFO was diagnosed.

Due to lack of clinical conditions such as thromboembolism, stroke, platypnea-orthodeoxia, migraine headaches, or practicing sport diving, which would make a PFO closure indicated, no interventions were recommended[3,4,5].

We report this casuistics to strengthen the clinical spotlight on the interatrial septum. If an ASA is apparent in TTE, it is obligatory to exclude a PFO, and in this case, we back up the hypothesis that contrast-enhanced TTE often possess high accuracy and economical benefit compared with TEE[6]. Therefore, a TTE should be the initial test to evaluate for the presence of an associated PFO.

### REFERENCES

1. Hoffman, J.I. and Kaplan, S. (2002) The incidence of congenital heart disease. *J. Am. Coll. Cardiol.* **39,** 1890–1900.
2. de Belder, M.A., Tourikis, L., Griffith, M., Leech, G., and Camm, A.J. (1992) Transesophageal contrast echocardiography and color flow mapping: methods of choice for the detection of shunts at the atrial level? *Am. Heart J.* **124,** 1545–1550.
3. Papa, M., Gaspardone, A., Fracasso, G., Ajello, S., Gioffre, G., Iamele, M., Iani, C., and Margonato, A. (2009) Usefulness of transcatheter patent foramen ovale closure in migraineurs with moderate to large right-to-left shunt and instrumental evidence of cardiovascular damage. *Am. J. Cardiol.* **104,** 434–439.
4. Dowson, A., Mullen, M.J., Peatfield, R., Muir, K., Khan, A.A., Wells, C., Lipscombe, S.L., Rees, T., De Giovanni, J.V., Morrison, W.L., Hildick-Smith, D., Elrington, G., Hillis, W.S., Malik, I.S., and Rickards, A. (2008) Migraine Intervention with STARFlex Technology (MIST) trial: a prospective, multicenter, double-blind, sham-controlled trial to evaluate the effectiveness of patent foramen ovale closure with STARFlex septal repair implant to resolve refractory migraine headache. *Circulation* **117,** 1397–1404.
5. Windecker, S. and Meier, B. (2008) Is closure recommended for patent foramen ovale and cryptogenic stroke? Patent foramen ovale and cryptogenic stroke: to close or not to close? Closure: what else! *Circulation* **118,** 1989–1998.
6. Zuber, M., Cuculi, F., Oechslin, E., Erne, P., and Jenni, R. (2008) Is transesophageal echocardiography still necessary to exclude patent foramen ovale? *Scand. Cardiovasc. J.* **42,** 222–225.

**This article should be cited as follows:**

Nia, A.M., Gassanov, N., Caglayan, E., and Er, F. (2010) Atrial septal aneurysm: a major diagnostic clue for a patent foramen ovale. *TheScientificWorldJOURNAL* **10**, 988–989. DOI 10.1100/tsw.2010.93.
